# Supplementary figures and images for: Epigenome and transcriptome changes in KMT2D-related Kabuki syndrome Type 1 iPSCs, neuronal progenitors and cortical neurons
Source: PLoS Genet. 2025 Sep 19;21(9):e1011608. doi: 10.1371/journal.pgen.1011608 (PMC12468740; doi:10.1371/journal.pgen.1011608)

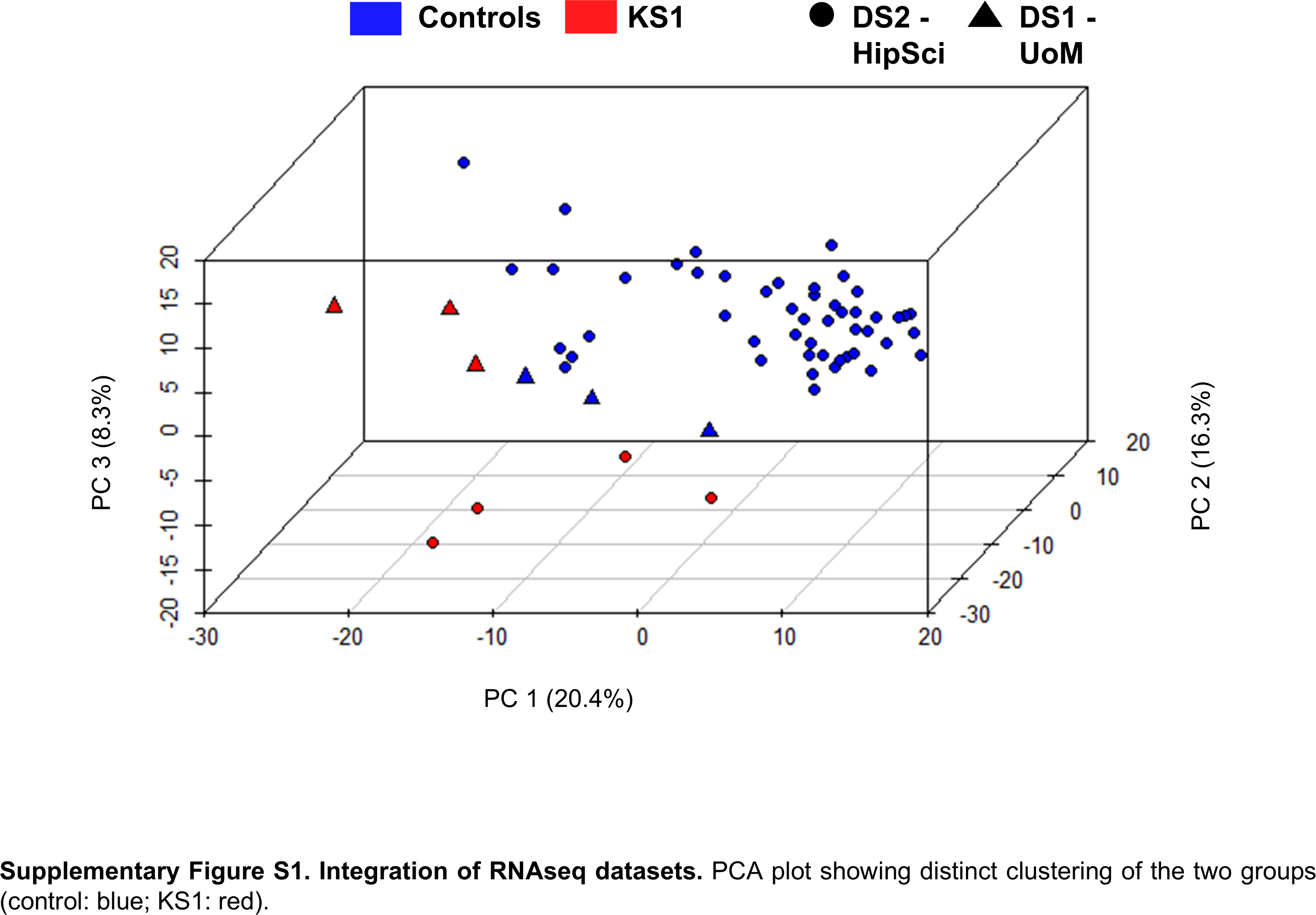

Supplement: S1 Fig — PCA plot showing distinct clustering of the two groups (control: blue; KS1: red). (TIF) [file pgen.1011608.s006.tif]

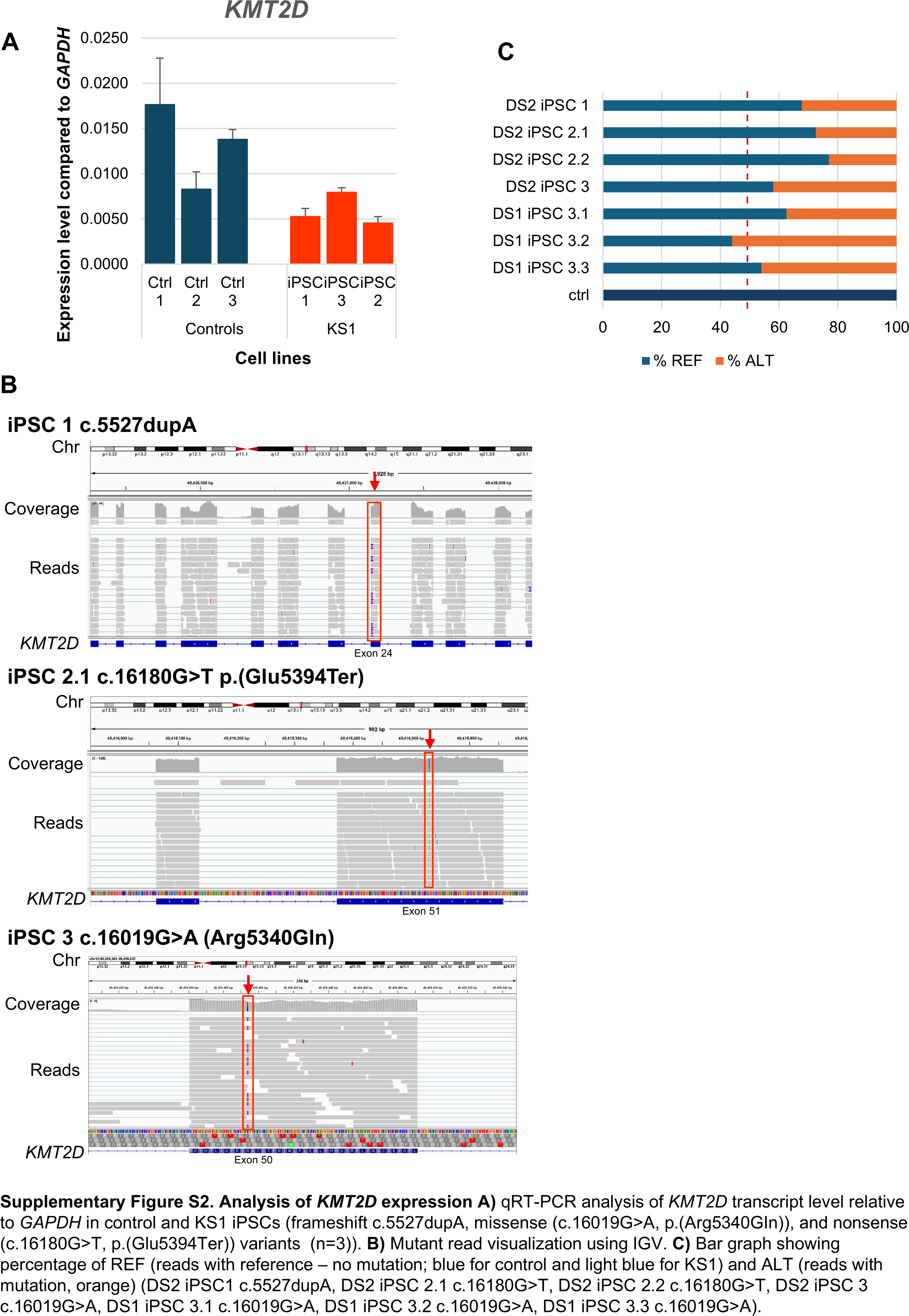

Supplement: S2 Fig — A) qRT-PCR analysis of KMT2D transcript level relative to GAPDH in control and KS1 iPSCs (frameshift (c.5527dupA, p. (Pro1849Ter)), missense (c.16019G > A, p. (Arg5340GIn)), and nonsense (c.16180G > T, p. (Glu5394Ter)) variants (n = 3)). B) Mutant read visualization using IGV. C) Bar graph showing percentage of REF (reads with reference – no mutation; blue for control and light blue for KS1) and ALT (reads with mutation, orange) (DS2 iPSC1 c.5527dupA, DS2 iPSC 2.1 c.16180G > T, DS2 iPSC 2.2 c.16180G > T, DS2 iPSC 3 c.16019G > A, DS1 iPSC 3.1 c.16019G > A, DS1 iPSC 3.2 c.16019G > A, DS1 iPSC 3.3 c.16019G > A). (TIF) [file pgen.1011608.s007.tif]

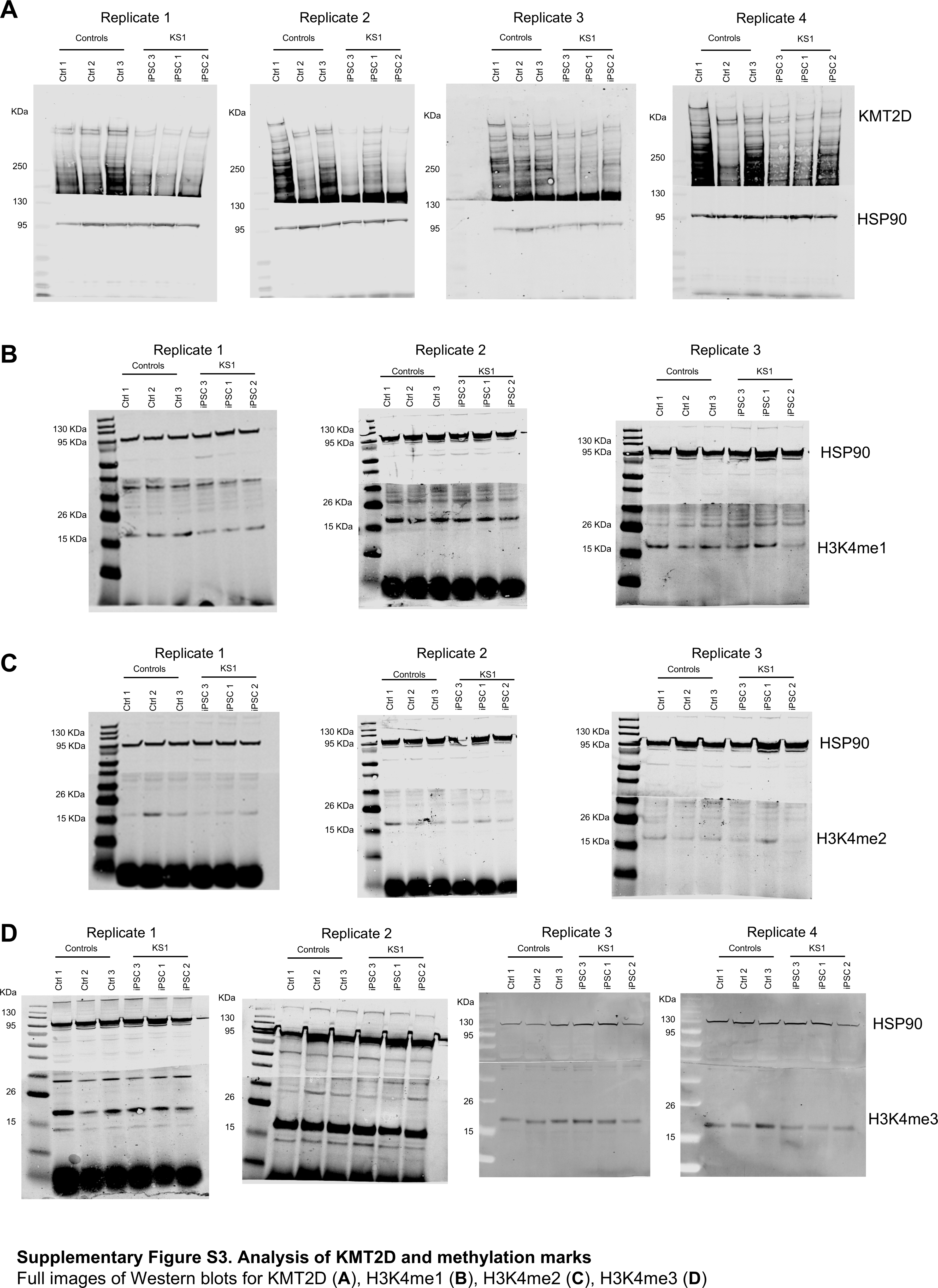

Supplement: S3 Fig — Full images of Western blots for KMT2D (A), H3K4me1 (B), H3K4me2 (C), H3K4me3 (D). (TIF) [file pgen.1011608.s008.tif]

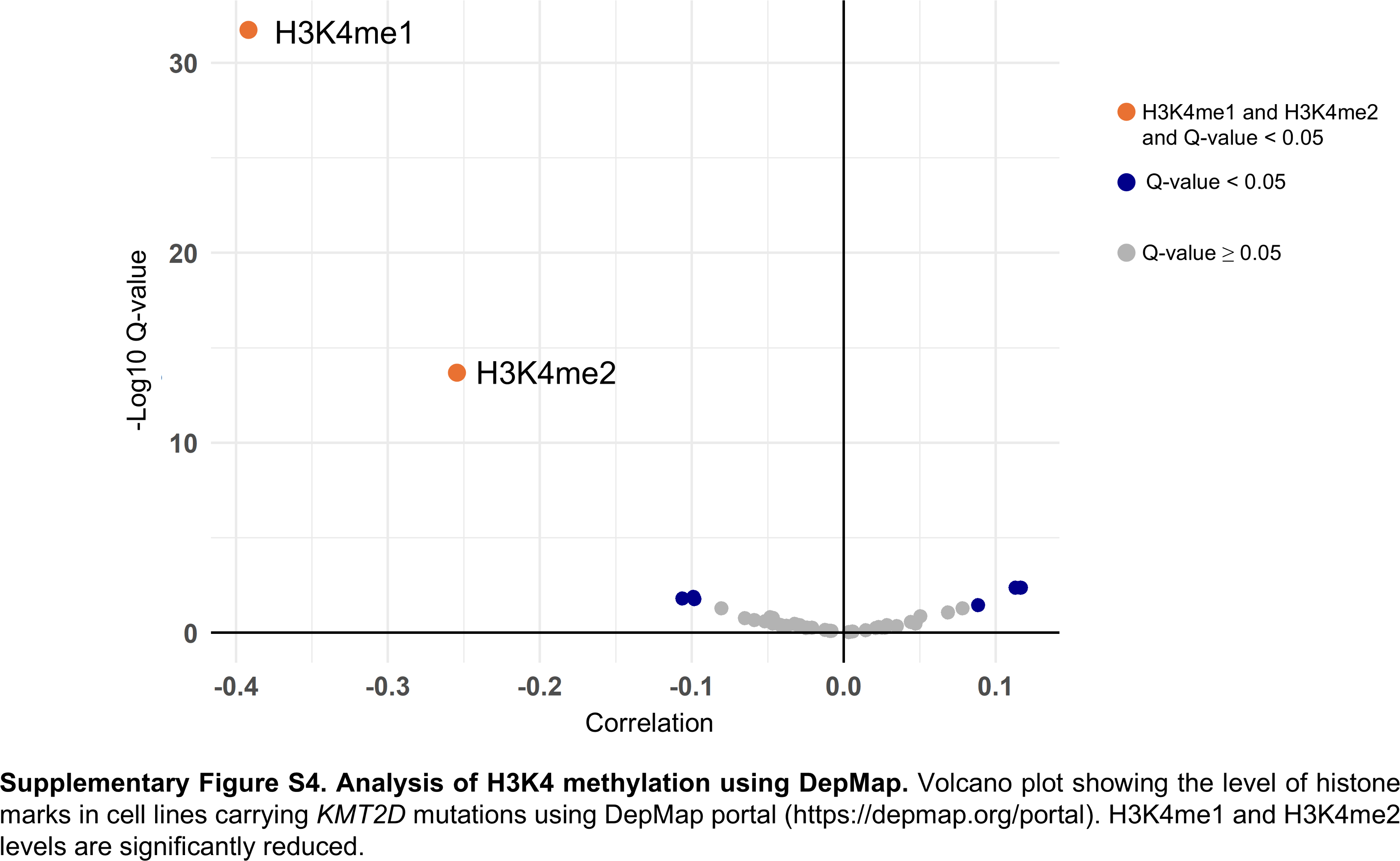

Supplement: S4 Fig — Volcano plot showing the level of histone marks in cell lines carrying KMT2D mutations using DepMap portal (https://depmap.org/portal). H3K4me1 and H3K4me2 levels are significantly reduced. (TIF) [file pgen.1011608.s009.tif]

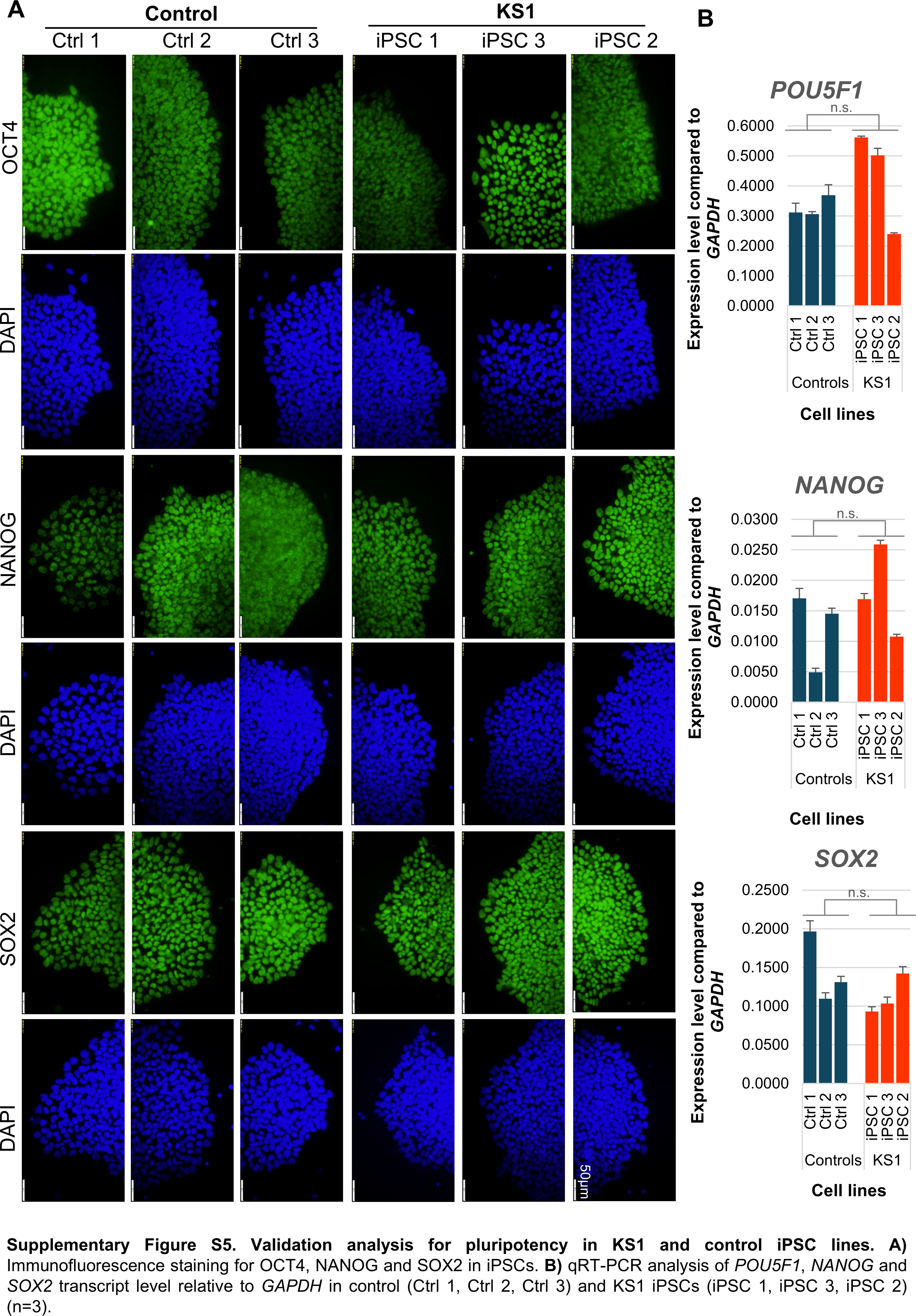

Supplement: S5 Fig — A) Immunofluorescence staining for OCT4, NANOG and SOX2 in iPSCs. B) qRT-PCR analysis of POU5F1, NANOG and SOX2 transcript level relative to GAPDH in control (Ctrl 1, Ctrl 2, Ctrl 3) and KS1 iPSCs (iPSC 1, iPSC 3, iPSC 2) (n = 3). (TIF) [file pgen.1011608.s010.tif]

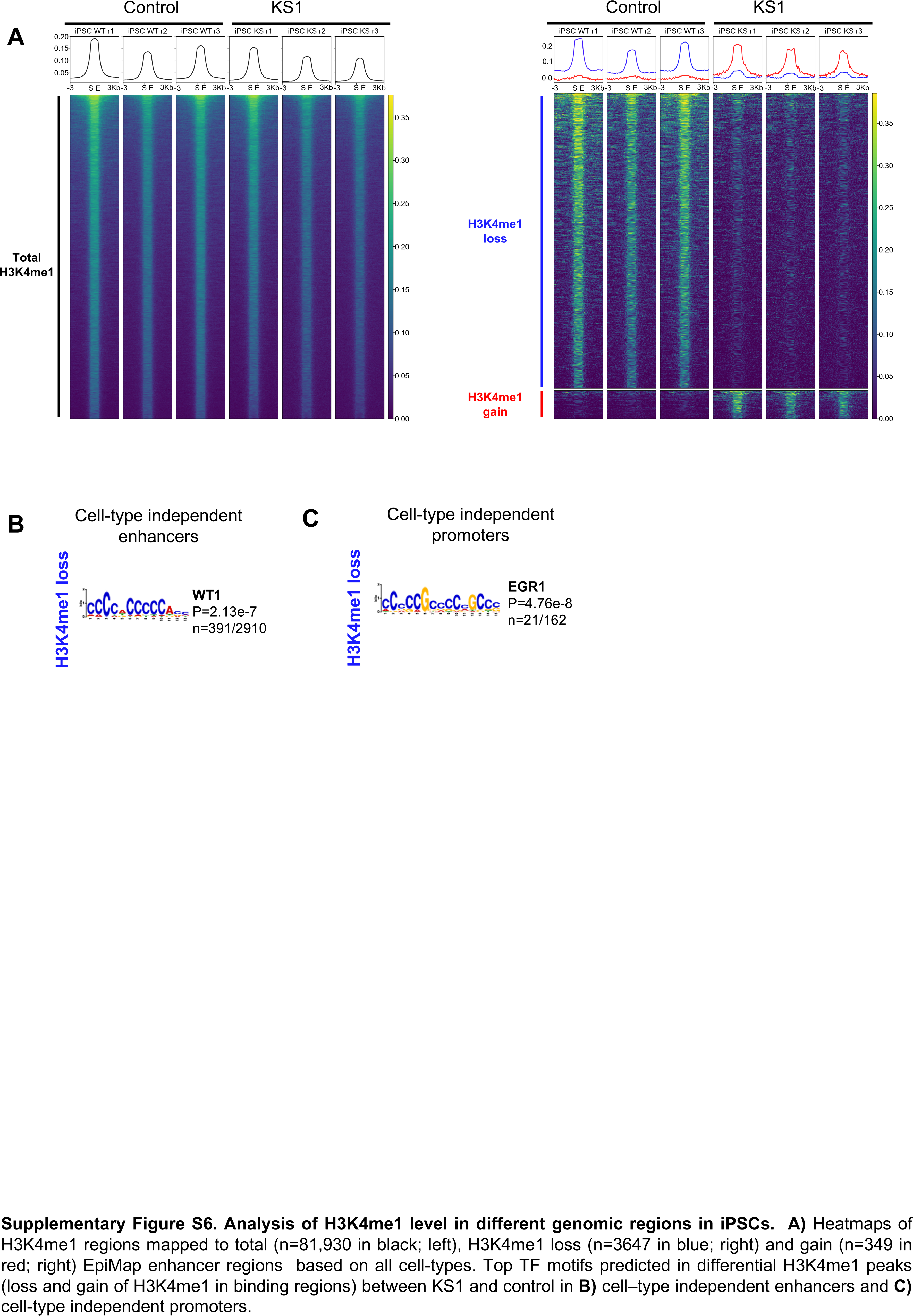

Supplement: S6 Fig — A) Heatmaps of H3K4me1 regions mapped to total (n = 81,930 in black; left), H3K4me1 loss (n = 3647 in blue; right) and gain (n = 349 in red; right) EpiMap enhancer regions based on all cell-types. Top TF motifs predicted in differential H3K4me1 peaks (loss and gain of H3K4me1 in binding regions) between KS1 and control in B) cell–type independent enhancers and C) cell-type independent promoters. Number (n) represents the number of times the motif was found within the unique sequences underlying the differential H3K4me1 regions. (TIF) [file pgen.1011608.s011.tif]

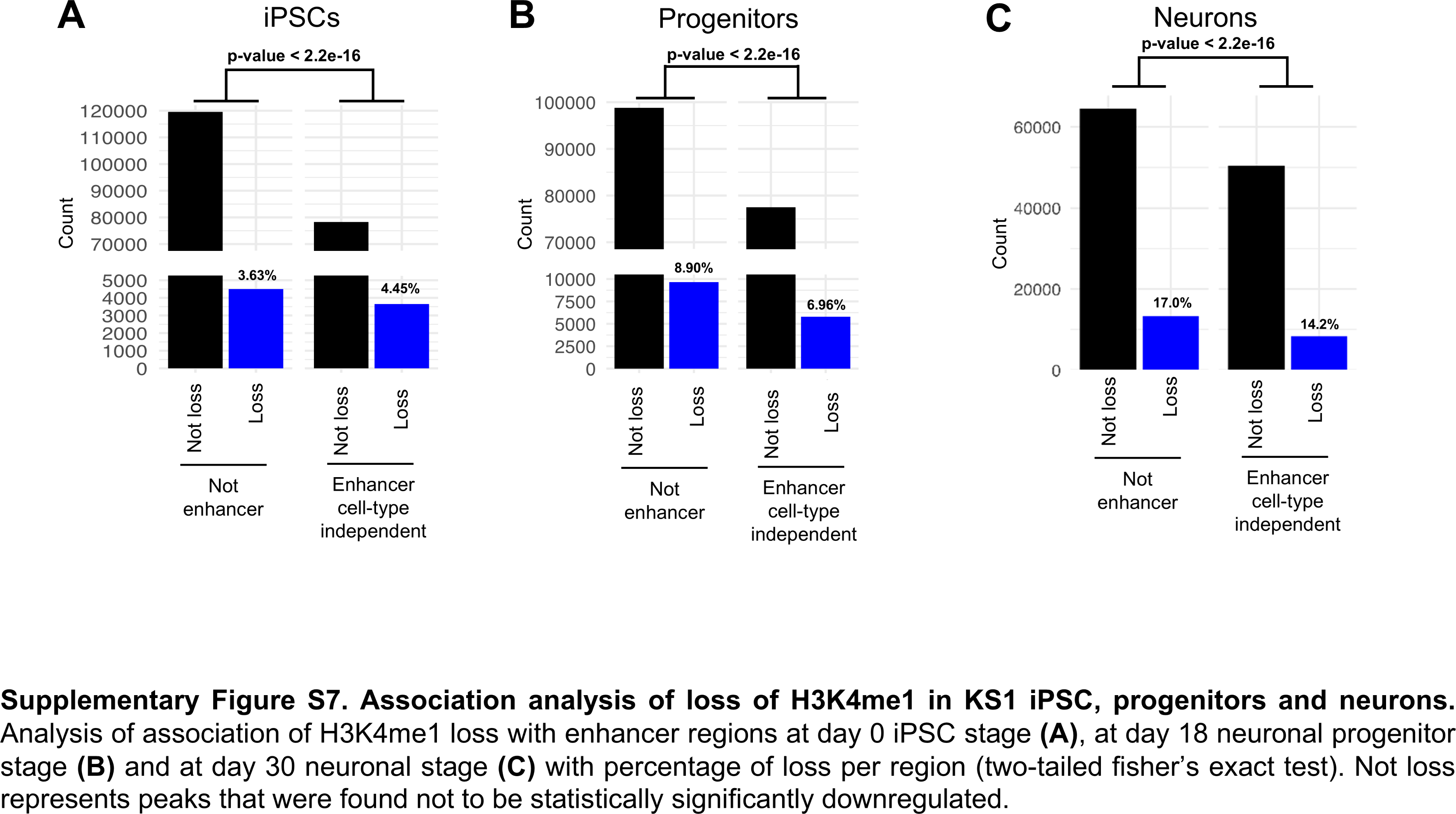

Supplement: S7 Fig — Analysis of association of H3K4me1 loss with enhancer regions at day 0 iPSC stage (A), at day 18 neural progenitor stage (B) and at day 30 neuronal stage (C) with percentage of loss per region (two-tailed fisher’s exact test). Not loss represents peaks that were found not to be statistically significantly downregulated. (TIF) [file pgen.1011608.s012.tif]

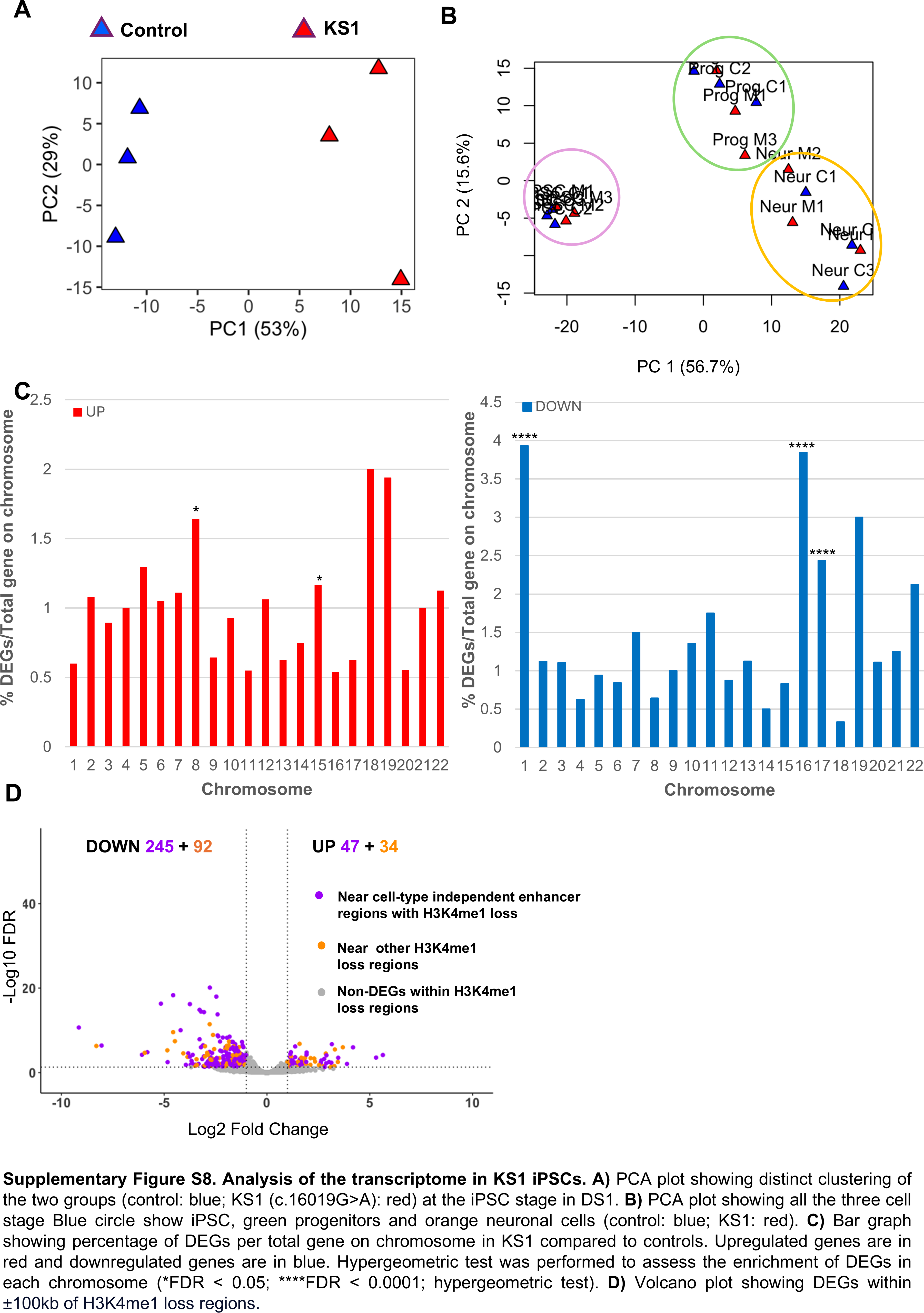

Supplement: S8 Fig — A) PCA plot showing distinct clustering of the two groups (control: blue; KS1 (c.16019G > A): red) at the iPSC stage in DS1-UoM. B) PCA plot showing all the three cell stage Blue circle show iPSC, green progenitors and orange neuronal cells (control: blue; KS1: red). C) Bar graph showing percentage of DEGs per total gene on chromosome in KS1 compared to controls. Upregulated genes are in red and downregulated genes are in blue. Hypergeometric test was performed to assess the enrichment of DEGs in each chromosome (*FDR < 0.05; ****FDR < 0.0001; hypergeometric test). D) Volcano plot showing DEGs within ±100kb of H3K4me1 loss regions. (TIF) [file pgen.1011608.s013.tif]

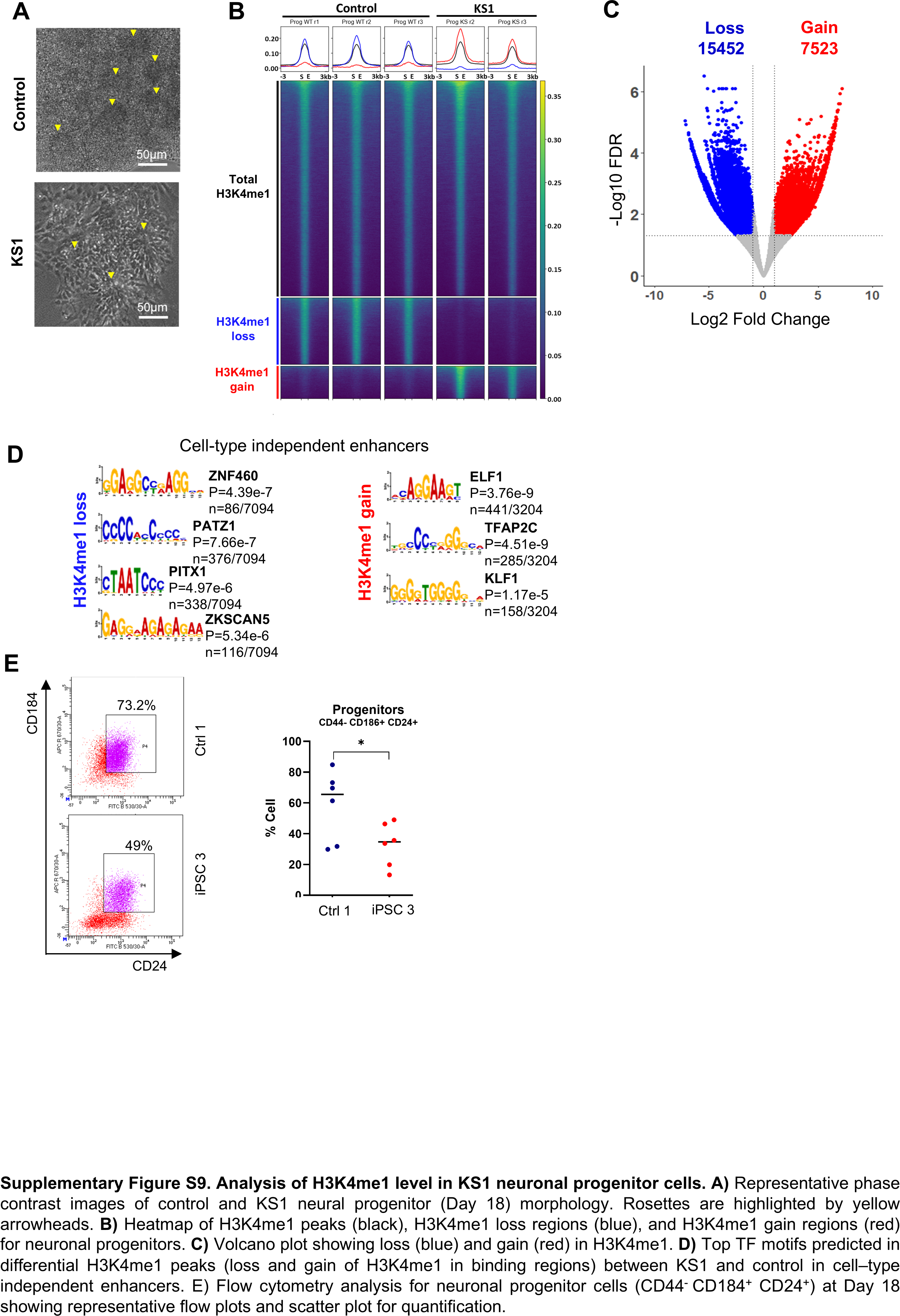

Supplement: S9 Fig — A) Representative phase contrast images of control and KS1 neural progenitor (Day 18) morphology. Rosettes are highlighted by yellow arrowheads. B) Heatmap of H3K4me1 peaks (black), H3K4me1 loss regions (blue), and H3K4me1 gain regions (red) for neural progenitors. C) Volcano plot showing loss (blue) and gain (red) in H3K4me1. D) Top TF motifs predicted in differential H3K4me1 peaks (loss and gain of H3K4me1 in binding regions) between KS1 and control in cell–type independent enhancers. Number (n) represents the number of times the motif was found within the unique sequences underlying the differential H3K4me1 regions. E) Flow cytometry analysis for neural progenitor cells (CD44- CD184+ CD24+) at Day 18 showing representative flow plots and scatter plot for quantification. (TIF) [file pgen.1011608.s014.tif]

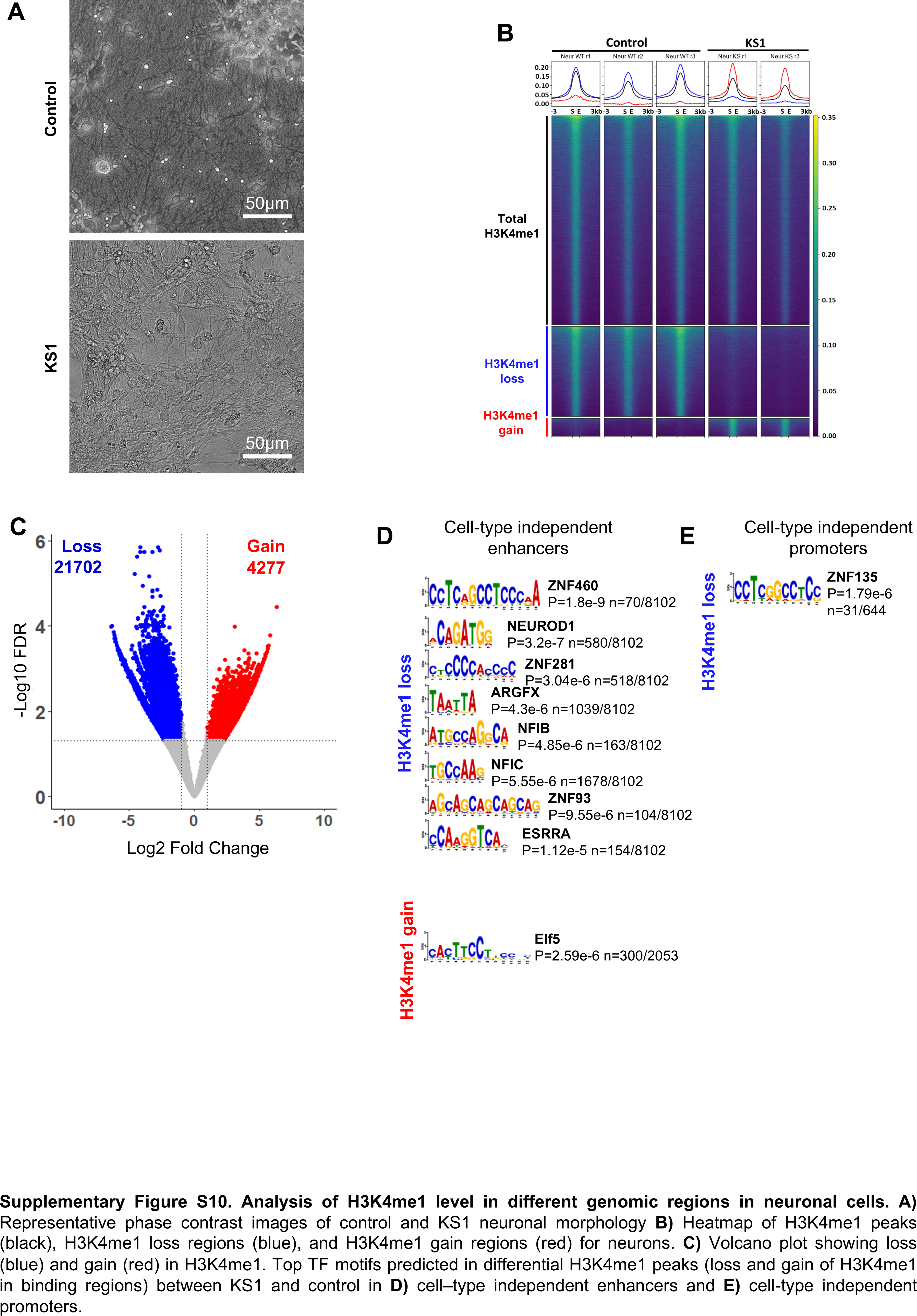

Supplement: S10 Fig — A) Representative phase contrast images of control and KS1 neuronal morphology B) Heatmap of H3K4me1 peaks (black), H3K4me1 loss regions (blue), and H3K4me1 gain regions (red) for neurons. C) Volcano plot showing loss (blue) and gain (red) in H3K4me1. Top TF motifs predicted in differential H3K4me1 peaks (loss and gain of H3K4me1 in binding regions) between KS1 and control in D) cell–type independent enhancers and E) cell-type independent promoters. Number (n) represents the number of times the motif was found within the unique sequences underlying the differential H3K4me1 regions. (TIF) [file pgen.1011608.s015.tif]

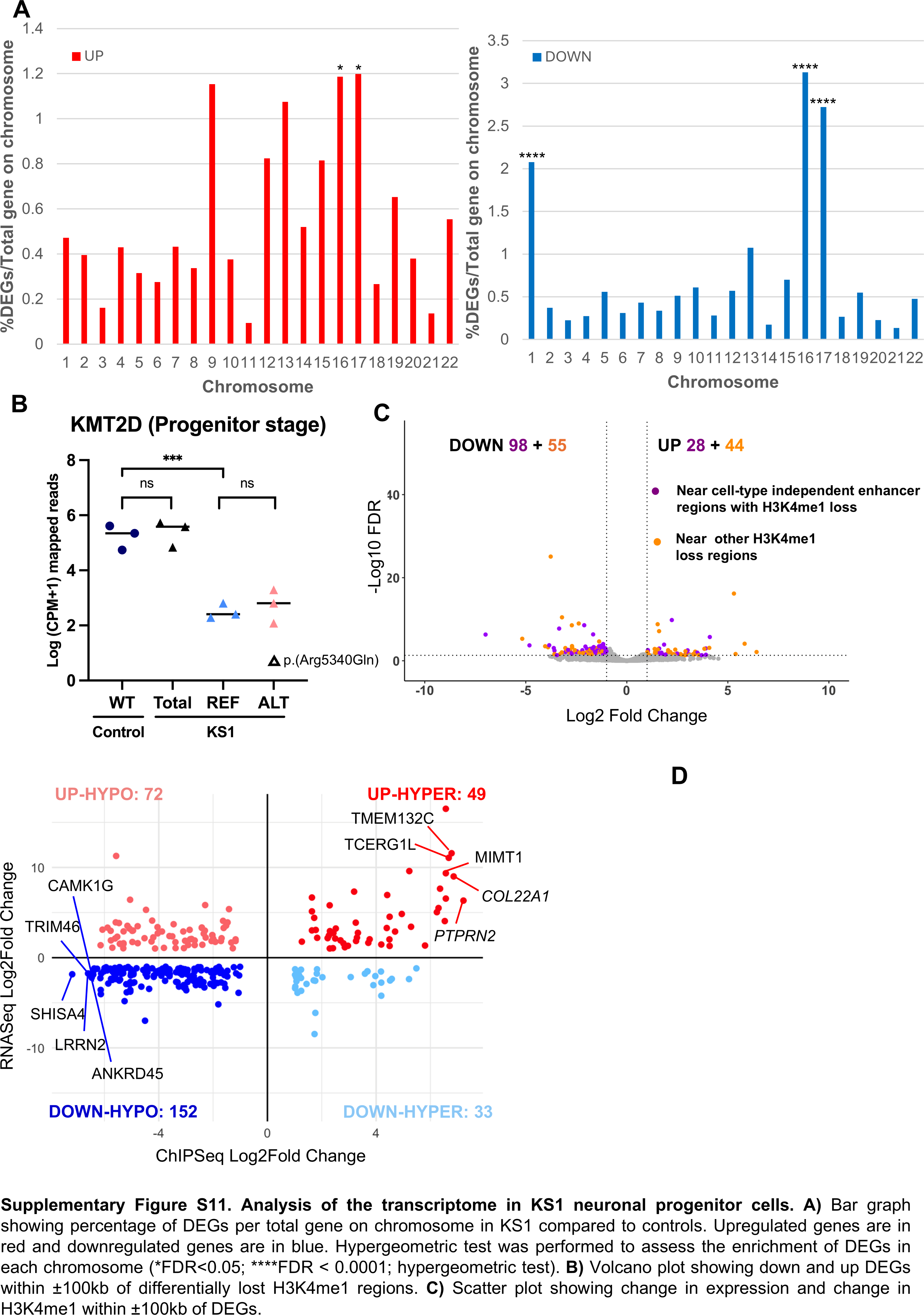

Supplement: S11 Fig — A) Bar graph showing percentage of DEGs per total gene on chromosome in KS1 compared to controls. Upregulated genes are in red and downregulated genes are in blue. Hypergeometric test was performed to assess the enrichment of DEGs in each chromosome (*FDR < 0.05; ****FDR < 0.0001; hypergeometric test). B) Volcano plot showing down and up DEGs within ±100kb of differentially lost H3K4me1 regions. C) Scatter plot showing change in expression and change in H3K4me1 within ±100kb of DEGs. (TIF) [file pgen.1011608.s016.tif]

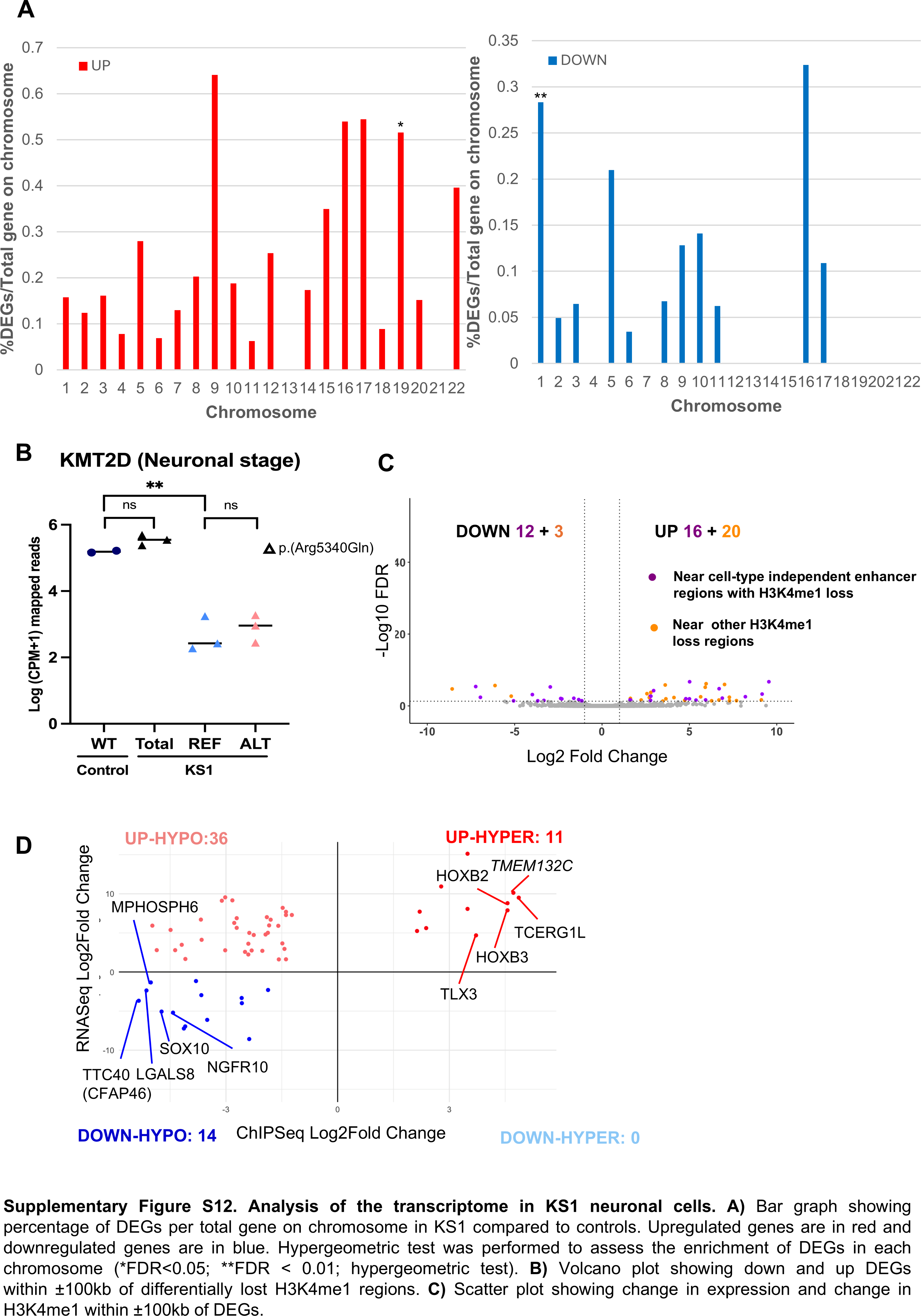

Supplement: S12 Fig — A) Bar graph showing percentage of DEGs per total gene on chromosome in KS1 compared to controls. Upregulated genes are in red and downregulated genes are in blue. Hypergeometric test was performed to assess the enrichment of DEGs in each chromosome (*FDR < 0.05; **FDR < 0.01; hypergeometric test). B) Volcano plot showing down and up DEGs within ±100kb of differentially lost H3K4me1 regions. C) Scatter plot showing change in expression and change in H3K4me1 within ±100kb of DEGs. (TIF) [file pgen.1011608.s017.tif]

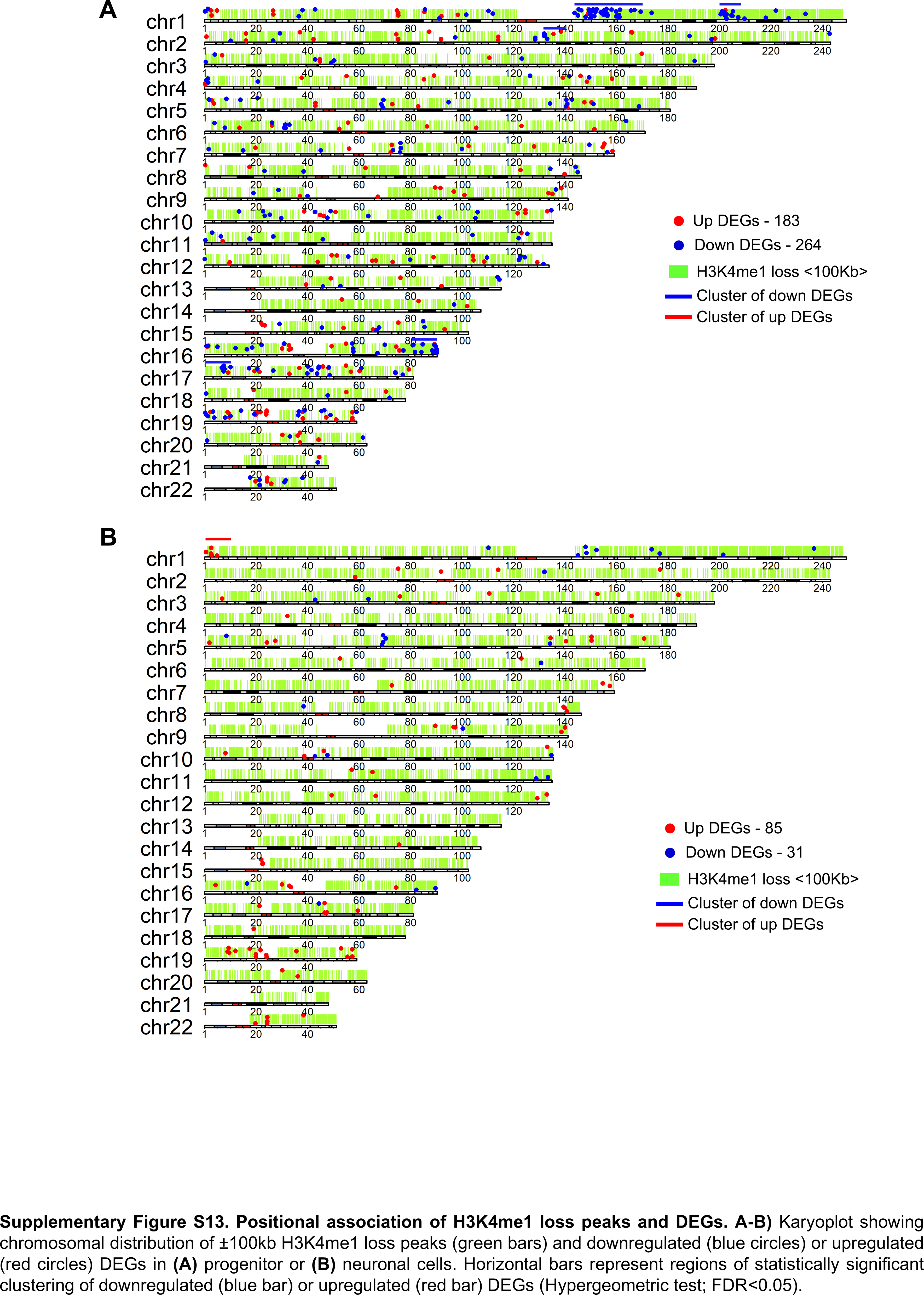

Supplement: S13 Fig — A-B) Karyoplot showing chromosomal distribution of ±100kb H3K4me1 loss peaks (green bars) and downregulated (blue circles) or upregulated (red circles) DEGs in (A) progenitor or (B) neuronal cells. Horizontal bars represent regions of statistically significant clustering of downregulated (blue bar) or upregulated (red bar) DEGs (Hypergeometric test; FDR < 0.05). (TIF) [file pgen.1011608.s018.tif]
