# Supplementary material for: Epigenome and transcriptome changes in KMT2D-related Kabuki syndrome Type 1 iPSCs, neuronal progenitors and cortical neurons
Source: PLoS Genet. 2025 Sep 19;21(9):e1011608. doi: 10.1371/journal.pgen.1011608 (PMC12468740; doi:10.1371/journal.pgen.1011608)
Supplement: Attachment — Submitted filename: Reviewers comments PLoS Genetics.docx [file pgen.1011608.s026.docx]

Response to Referees’ comments

Epigenome and transcriptome changes in KMT2D-related Kabuki syndrome Type 1 iPSCs, neuronal progenitors and cortical neurons

We thank the referees for their constructive comments. All changes have been added and highlighted in red and deleted text is tracked throughout the manuscript.

**Reviewers' comments:**

**Reviewer #1:** Cuvertino et al., perform a comprehensive mechanistic assessment of changes in gene expression and histone methylation across iPSC, neuronal progenitor and neuronal cell types. They address how changes in histone methylation may underlie the traits associated with the neurodevelopment Kabuki Syndrome. Overall, this study is very interesting and bridges an important gap in the knowledge as to the underlying molecular mechanisms of Kabuki syndrome.

We thank the reviewer for their positive comment.

I suggest the following changes to the test to improve this paper:

1) The authors state that the number of differential H3K4me1 peaks between KS1 and control samples is reduced through differentiation and conclude that this reflects a difference in the requirement of KS1 during the differentiation process. An alternative interpretation is that chromatin compaction increases with differentiation, as cells become more specialised and only express genes required for their lineage, as opposed to the more open and permissive chromatin state in iPSCs and stem cells. As such, the number of regions able to be methylated on H3K4 will be reduced as cells differentiate, and this may underly the reduced number of H3K4me1 peaks in the progenitors and neurons in this study, relative to the iPSCs.

We agree with the reviewer’s line of thought, that as cells become more specialised, number of H3K4me1 peaks should decrease. Indeed, this is exactly what we find (see peaks in control cell line in **Figures 2C**, **4E** and **F**). However, we find the number of differential H3K4me1 peaks between KS1 and control increased. This observation of a proportion of differential H3K4me1 peaks increasing with differentiation makes our results even more striking.

2) Do the KS1 and control iPSCs differentiate into neurons with different efficiencies? The images in Supp Figure 8 show that they have markedly different morphologies at day 18. A comment on the efficiency of neuronal differentiation across KS1 lines relative to controls should be added to the results or discussion.

We observed a decrease in number of KS1 progenitors at Day18. These data are now included in **Supplementary Figure S9E**. A comment regarding this has also been included in the discussion **(page 11, line 390-392)**.

3) Supplemental Table 1 shows that the iPSC control lines are from 2 male donors and 1 female, while the KS1 samples are from 2 females and 1 male. The potential for sex-associated differences across datasets should be discussed. To this point, iPSC line 1 and 3 show much higher levels of Oct4 and Nanog expression by RTqPCR, compared to iPSC line 2. iPSC lines 1 and 3 are female, while iPSC line 2 is male, such that sex-specific differences may underlie this result. Although, this could also be explained by iPSC line 2 carrying a nonsense mutation rather than a missense or frameshift as in the other two lines. Regardless, a discussion on potential sex differences should be added to the discussion and I suggest that the authors reword their comment about pluripotency gene expression not being different in these cells as it is markedly increased at Oct4 and Nanog in 2 of their 3 cell lines.

We thank the reviewer for raising this important point. Previous research (Pottmeier *et al.* 2024) found no differences in expression of NANOG and OCT4 with sex in human embryonic stem cells (5 females and 3 males) by assessing gene counts from RNASeq. As suggested we have added reference to difference in sex of the cells used here in the discussion **(line 365)**. Regarding the comment about pluripotency gene expression, please note that the transcript levels for *OCT4* and *NANOG*, analysed by qRT-PCR, are not statistically different between our lines. This has now been added in the bar graph in the **Supplementary Figure S5.**

4) I would suggest replacing Figure 1 B with Supplemental Figure 2A. Supplemental Figure 2A is a bit easier to interpret.

We thank the reviewer for this suggestion. **Figure 1B** illustrates an important point by showing the significant difference in levels of mutant (ALT) and REF transcripts, which is not obvious when comparing the overall gene expression values as shown in **Supplementary Figure 2A**. We, therefore, would like to retain Figure 1B as a main figure. However, to improve clarity we have edited the key, the figure legend and the relevant sentence in the main text **(line 110-111)**.

5) Which control iPSC line(s) were used in ChIP-seq experiments? The authors do not specify and whether this was a male or female line would be relevant to add to the discussion.
We thank the reviewer for raising this important point. The sex of all lines has now been provided in the new **Supplementary Table 1**.

6) Were the regions with gained H3K4me1 in the ChIP-seq datasets notable in any way? A comment should be added to the results section.

We thank the reviewer for raising this point. We indeed made several notable observations in our ChIP-Seq dataset, as already mentioned in the original manuscript. Below we have also summarised these observations for ease of your reference.

- In KS1 iPSCs 1,163 sites showed gain in the H3K4me1 peak signal (**Figure 2A-C**) **(Line 147**).
- KS1 iPSC sites that gained H3K4me1 peaks showed significant enrichment for KLF1 transcription factor binding site (**Figure 2D**) (**Line 157**).
- In KS1 neural progenitors 7,523 sites showed gain in H3K4me1 peak signal (**Figure 4C-4E** and **Supplementary Figure S10**) (**Line 224**).
- In neurons, 4,272 sites showed gain in H3K4me1 peak signal (**Figure 4D-4F** and **Supplementary Figure S11**) (**Line 226**).
- Motif analysis on neuronal progenitors revealed that the sites where H3K4me1 was gained were predicted to be targets for IKZF1, KLF4, FOXO6 and TFAP2C (**Figure 4G**). ELF1, TFAP2C and KLF1 with cell-type independent enhancers with H3K4me1 gain (**Supplementary Figure S9D**) (**Line 233**).
- the comparison of the significantly differentially methylated peaks across the three stages of neuronal differentiation identified 88 H3K4me1 regions (74 losses and 14 gains) that were affected across all stages in the KS1 lines (**Figure 4I**) (**Line 244**).
- In neural progenitors and neurons, we found statistically significant association between upregulated DEGs and H3K4me1 gain at distances 5kb to 100kb between the H3K4me1 peak and gene TSS (hypergeometric test; p-value < 0.05) (**Supplementary Table 4**) (**Line 279**).

7) Supplemental files with the differentially expressed genes and regions of differential H3K4me1 abundance across datasets should be provided. Also, it is not clear if these datasets will be uploaded to public platforms.

We completely agree with the reviewer regarding data availability. The Datasets are deposited on GEO with restricted access until publication. We have also now included a new file (**Supplementary information file**) that lists all the DEGs and H3K4me1 differentially methylated peaks.

**Reviewer #2:** This manuscript explores the transcriptomic and epigenomic mechanisms underlying Kabuki syndrome 1 (KS1) in three KS1 patient-derived induced pluripotent stem cells (iPSC) lines. This would be a tremendous resource for the neurodevelopmental disorder field and further our understanding into disease mechanisms of KS1. The authors have addressed questions relevant to KS1 and conducted appropriate experiments to validate their iPSC models in relation to current knowledge of KMT2D function. It’s evident that the overarching goal of this study was to compare and contrast the transcriptome and epigenome of different KS1 mutations across pluripotent stem cells and more disorder-relevant cell types including neural progenitors and mature neurons. In its current state, this manuscript falls short of these aims and lacks sufficient detail and clarity regarding the datasets used between experiments and rationale for focusing primarily on the missense variants in neural cell types (detailed below). This reviewer recommends the following major corrections:

We thank the reviewer for their positive comments about the importance of this work and our experimental design. In the revised version, we have attempted to provide more details and improve clarity and justify our selection of the cell line for more detailed work.

**Clarity around datasets, controls, replicates and lines**

1) Why were different datasets used for experiments and why do some iPSC line appear across multiple datasets but others do not.

The new **Supplementary Table 1** provides details regarding the lines with information on mutations, number of clones and biological replicates.

The data presented in the manuscript includes

- RNASeq dataset previously generated on 50 controls and 3 mutant lines (1 line had two clones tested) by Hipsci. This was used only to compare KMT2D expression **(Figure 1B)** and pluripotency genes expression **(Figure 1E)**.
- Please note HipSci does not provide relevant ChIPSeq data or RNASeq data at progenitor and neuronal stages. Therefore, for maintaining methodological consistency to track the transcriptomic changes we only used the data generated in our lab with our hands for results shown in Figures 2A to 5F.
- As these experiments were performed only on one control and one missense mutation line we wanted to validate the results in additional lines. Therefore, results shown in **Figure 5G-H** were generated using additional control and KS1 lines differentiated up to D18.

Ideally, we would have liked to perform all the experiments across multiple stages in multiple cell lines, which was unfortunately beyond the scope due to the resource limitations and time constraints.

2) Additional details regarding the control lines used is required (are they isogenic corrected or from healthy individuals).

More details about the cell lines are now provided in the new **Supplementary Table 1**. iPSC control lines were generated from healthy individuals.

3) There are 6 controls listed in supp. Table 1 across three datasets, are these all from different individuals or are some of these matching? This needs to be stated.

More details about the cell lines are now provided in the new **Supplementary Table 1**

4) What denotes an iPSC line vs a replicate? It appears multiple lines/replicates have been generated from a single individual. In it’s current state, this is confusing and lacks sufficient detail to interpret the results sufficiently. Are lines generated via an independent induction experiment or are they different clones from the same experiment? Are replicates internal replicates or experimental replicates?

iPSC lines were generated from different donors. The nonsense Kabuki iPSC line (iPSC2) generated two different clones. Replicates are experimental replicates. Specifically, Ctrl 1 and iPSC 3 were differentiated into cortical neurons three different times (experimental replicates) and used for RNAseq and ChIPseq experiments. This has also been clarified in the manuscript and in the new **Supplementary Table 1**.

5) If the author is merging independent biological replicates and experimental replicates for analysis, how are they accounting for these variables statistically?

We only used merged datasets (generated by HipSci and our lab at the University of Manchester) for the quantification of pluripotency genes and *KMT2D* gene expression. For the differential analysis, we have not used the merged datasets so linear modelling accounting for two datasets was not performed.

**Discordance in experiments across the study**

6) The authors initially explored the impact of three Kabuki Syndrome 1, KMT2D patient variants – a missense, a nonsense and frameshift variant – on H3K4 methylation and KMT2D expression in pluripotent stem cells. The remainder of the experiments in this study were less powered and only focused on the missense mutation. It is unclear why the authors did not pursue a comparison of different KS1 mutations across the experiments in this manuscript and why the missense mutation was chosen for further analysis, especially in light of the differing mRNA results in Fig 1B which may allude to a differing mechanism and impact on transcription and H3K4 methylation in comparison to other mutations.

We appreciate your constructive feedback. H3K4 methylation and KMT2D expression in pluripotent stem cells are relatively less resource demanding, and therefore were performed on all available cell lines. As expected, these results showed that the missense line has less effect on mRNA levels of KMT2D, but the downstream consequences on KMT2D protein levels and its product H3K4me1 were same in all the three cell lines. This indicates that although the immediate molecular mechanism is different, the immediate consequences of all the mutations studied here are likely to be identical (i.e. decrease in protein expression and decrease in overall H3K4me1 levels). We chose to perform the main experiments on single mutant and control lines to manage the project within available resources. Even though ~10% KS1-causing variants are missense, most previous studies have used models with null variants. This was another motivation to choose the missense variant line. We agree that performing these experiments on all cell lines would have been ideal. However, resource constraints and time limitations did not allow. As pointed out by the reviewer, we attempted to overcome this using validation experiments.

7) The authors partially address this in the last section by conducting an additional validation RNAseq experiment with two control lines and the frameshift variant in neural progenitors. DEG comparison with the missense variant experiment had a moderate positive correlation, suggestive of a similar trend in gene expression changes between the missense and frameshift mutations. The nonsense mutation is however absent from this analysis and is only included within the first RNAseq experiment in iPSC’s.

The Validation RNAseq dataset in **Figure 5G** was performed using all 3 KS1 mutations (**Supplementary Table 1**). This information is now included in the Figure legend (**line 660**). Please also see the new **Supplementary Table 1**.

8) The authors should consider changing the focus of this study to primarily reflect their experiments which are concentrated on assessing transcriptomic and epigenetic differences in the missense mutation in different cell types in comparison to healthy control lines to improve the clarity and comprehension of the manuscript.

We have clarified and improved the manuscript specifying that the focus of this study is on assessing transcriptomic and epigenomic differences in the missense mutation.

Minor corrections:

Figure 1

9) Western blot quantification graphs in panel C and D need units for the Y-axis.

The units for Y-axis now added in the relevant figure panels.

10) In panel B, the graphical representation comparing ALT KMT2D transcripts between missense, nonsense and frameshift mutations and the associated significant bar is confusing and separating these into their genotypes would be helpful to better interpret the results.

We thank the reviewer for the suggestion. Please see the updated **Figure 1B** and associated legend.

11) In the figure legend, you need to state the statistical analysis conducted and the n for each group.
We have added the samples size and the statistical method used in the figure legend (**line** **593**).

12) Panel E: It’s unclear how these pluripotency markers were chosen. A geneset enrichment analysis with a pluripotency geneset would be more appropriate.
Supp. Table 1.

The pluripotency genes were selected from gene ontology pluripotency marker category. This has also been included in the material and methods section (**line 501**).

13) Having a combined column for # Samples/Sample name for Controls is confusing and should be separated. It is unclear whether controls from dataset 1 and 2 come from Ctrl 1, 2 or 3 individuals or different individuals. This would be more clear if the columns were separated and this information provided

We thank for the suggestion and have now clarified the information included in the **Supplementary Table 1**.

14) Number of samples should be in the sex column of DS2 controls.

As stated in the point above, we have now amended the **Supplementary Table 1** making it clearer.

15) Line 186: SUZ12 was identified only in DEGs from the RNAseq dataset which were upregulated, but not in downregulated peaks. Additionally you did not identify SUZ12 in your motif analysis at H3K4me1 peaks. This should be more accurately reflected in your summary of the data from these experiments.

SUZ12 is a component of Polycomb-repressor complex 2 (PRC2). PRC2, as well as SUZ12, do not bind directly to DNA (Cao *et al*. 2004), therefore, there are no PRC2/SUZ12-specific DNA motifs. We have emphasised the association of SUZ12 with the upregulated gene set (**line 203**).

Figure 3.
16) It is unclear why the authors have chosen to perform a DEG analysis on just the missense variant iPSC lines and not the other variants, especially considering they have these datasets based on the Fig 1. results. It would be interesting to conduct a DEG analysis across mutants in comparison to the control lines to determine if common or different genes and gene sets are dysregulated.

We thank the reviewer for the suggestion. Our intention was to track the differences in the expression data through the neuronal differentiation stages. As the D0 RNASeq data for other lines was generated by HipSci using a different protocol, we were unable to incorporate these into our analysis. D18 DEGs overlap analysis is already presented in **Figure 5G**.

17) Line 191: A better rationale for assessing neural progenitors is needed beyond stating KS1 clinical phenotypes.

We have included text explaining the association between changes in the neural progenitor population and neurodevelopmental disorders (**line 209**). We have also included an additional reference (number 19).

18) Line 224: these numbers should accurately reflect the numbers in Fig. 4I.

We have updated the numbers in the manuscript (**line 245**).

Figure 4.

19) In the figure legend an independent description of what cell types panel E and F represent should be provided.

We have added information on cell types used to impute enhancer regions for **Figure 2C** (**lines 609-611**); and for **Figure 4E-F** (**lines 637**).

20) The total loss peaks and gain peaks for each cell type in panel I do not match the numbers in panel E and F. For example, in panel I the total loss peaks for neurons is 21,584 (20321+402+74+787), but in panel F the total loss peaks is 21,586.
This discrepancy is due to the way the intersected peaks are calculated. As peaks can be of different sizes, some peaks in one set can intersect more than one peak in the other set. The R package used (ChIPPeakAnno) takes the smallest number of intersections, i.e., several peaks intersecting one peak would count as one intersection. This has been referred to previously and has been mentioned by the developer of the package: <https://support.bioconductor.org/p/62739/>.

21) Abbreviations such as Neur and Prog should be defined in the figure legend.

Abbreviations have been defined in the figure legends.

Figure 5.

22) To be more comparable with the iPSC datasets, similar representation of analyses should be displayed. Therefore A karyoplot like in Fig 3C should be included for neural progenitors and neurons.

We have included the karyoplots for neural progenitor and neuronal stages in **Supplementary** **Figure S13**. A paragraph has been added in the result section to describe the analysis (**line 277**).

23) Abbreviations such as Neur and Prog should be defined in the figure legend.
Abbreviations have been defined in the figure legends (**lines 641**, **653-656**).

24) Line 266: ‘into’ should be ‘in’

This has been corrected.

25) Supp Table 1 refers to 3 datasets – DS1, DS2 and DS3, yet in the text you also refer to a validated dataset (line 280 and 604 for example) which is confusing. This need to be clarified and added to the Supp Table 1.

We have clarified the information included in **Supplementary Table 1** and removed the DS1-3 labelling.

26) Line 313: The authors need to clarify that this was only demonstrated for the missense mutation.

We have clarified this (**line 400**).

27) Line 356: This is limited to the missense mutation based on the results from this study. Discussion around how the results of the missense mutation across cell types may inform on the mechanisms of the frameshift and nonsense variants in neural tissue and genomic H3K4me1 would be more accurate.

We have included a sentence regarding this in the discussion part (**line 406**)

**Reviewer #3:** This is an interesting study evaluating transcriptomic and epigenetic changes in iPSC-derived cell models from Kabuki syndrome type 1 (KS1) patients. The authors track the epigenome and transcriptome across three neuronal differentiation stages, identifying significant disruptions in KMT2D expression, H3K4 methylation, and enhancer-associated histone marks. They report that epigenomic alterations accumulate during differentiation, while the number of differentially expressed genes decreases. The study highlights the role of Polycomb-mediated repression and transcription factor binding changes in KS1 pathogenesis.
However, some issues regarding the generalizability of the data across different iPSC lines need to be addressed to enhance the study’s impact and clarity before publication.
We thank the reviewer for their positive comments about this work. In the revised version, we have attempted to provide more details on data generation and improve clarity throughout the manuscript.

Specific Comments

1) In this study, the authors performed transcriptomic and epigenetic profiling of iPSC-derived cell models (iPSCs, neuronal progenitors, and neurons) to investigate molecular changes in KS1. They utilized three different iPSC lines from KS1 patients. However, the core analyses were conducted using only one KS1 iPSC line with a missense mutation (c.16019G>A, p.(Arg5340Gln)), making it difficult to generalize the proposed mechanism (Polycomb-mediated repression and transcription factor binding changes). This issue is compounded by the lack of quality control data for the iPSC lines.

a. Figures 2–5: It is unclear what control was used in the comparison with the c.16019G>A (p.(Arg5340Gln)) line—was it a single control line or all controls?

We have used one control line in triplicate. This is also mentioned in the manuscript (**line 144-145**) and included in the new **Supplementary Table 1**.

b. iPSC Quality Control: There is no data on the quality control (e.g., karyotyping) of the KS1 iPSCs, which is essential for ensuring the reliability of the findings.

Cells were cultured in optimal condition and tested for pluripotency markers as shown in **Supplementary Figure S4**. Karyotyping of Ctrl 1, iPSC 1, iPSC 2 and iPSC 3 have been analysed by the HipSci consortium and we can be viewed on <https://www.hipsci.org/#/cohorts/kabuki-syndrome> for KS1 lines and <https://www.hipsci.org/#/lines/HPSI0314i-wigw_2> for the control line. Cells included in the experiments in this manuscript have always been used within 10 passages from the karyotyping analysis as per gold standard iPSC culture rule.

c. Figure 5G: The overlap of differentially expressed genes (DEGs) between the three KS1 iPSC lines is only briefly explored, and only between iPSCs and neuronal progenitors. The observed overlap between all three lines is of high interest, as these likely represent the most robust KMT2D-dependent changes. However, the presented data are sparse and feel incomplete, with no corresponding methylation data.

We agree that the overlap between the three KS1 variants is of high interest. Indeed, this is the aim for our future work. In the current manuscript, most of the molecular changes in KS1 were observed at the early stages of the neuronal differentiation therefore, we have analysed the two additional KS1 variants at the progenitor stage (Day 18). We have now included gene ontology analysis (**Figure 5H**) performed using the DEGs that overlap between the three KS1 lines at Day18 and description of the result in the manuscript (**line 311**).

2) Although several mechanisms are being proposed and discussed there no single validation presented.

We have added a sentence about the need to validate the proposed mechanisms (**line 342**).

3) Figure 1D: The authors should use H3 as a loading control to provide a more accurate estimation of H3 methylation changes. The number of technical and biological replicates for the western blot is unclear—does "N=3" refer to three independent iPSC lines, or does it mean the western blot was performed once for each line?

Hsp90 can be used as a nuclear control in Western blotting to assess protein loading. In the future H3 will be used. The western blot experiments were performed three independent times using the three controls and the three KS1 lines. Therefore, N=3 refers to three independent Western blots.

a. HSP90 Blot (H3K4me3): The blot appears problematic, as the protein seems to have been retained in the wells instead of migrating through the gel.

We have now included the full western blot images in the **Supplementary Figure S3**. This should clarify the doubts regarding protein migration through the gel.

b. There is considerable variation in the western blots that is not reflected in the quantification. How do the authors explain this? They should provide full western blot images for clarity.
The western blot experiments were performed three independent times using the three controls and the three KS1 lines. We have then quantified and shown the average of protein expression per samples. The western blot images included in the manuscript are a representative one. The full Western blot images are included in the **Supplementary Figure S3**.

Minor Comments

4) Figure 1B: The description in the text is unclear. Please rephrase for better comprehension.

We have amended the Figure 1B legend and the **Figure 1B** to make it clearer.

5) Figure 1C: Why would a missense mutation lead to decreased KMT2D levels? Do the authors have any explanation?

The decreased KMT2D levels in cells from samples with missense variants may be due to decreased protein stability. Indeed, previous analysis of KMT2D:p.(Arg5340Gln) variant showed that this variant reduces KMT2D protein stability (Cocciadiferro et al. 2018). We amended the discussion section to emphasise further on this previous finding (**lines 337-339**).

6) Figure Presentation: The manuscript, including figures, appears somewhat sloppy. Figures are not always clear, and their presentation could be improved.

We have improved the quality and presentation in our figures.

7) Figures 5 & 6: These figures are not referenced in the text and should likely be converted into tables for clarity.

These figures have been converted into tables.

**Have all data underlying the figures and results presented in the manuscript been provided?**
Large-scale datasets should be made available via a public repository as described in the *PLOS Genetics* [data availability policy [track.editorialmanager.com]](https://urldefense.com/v3/__http:/track.editorialmanager.com/CL0/http:*2F*2Fjournals.plos.org*2Fplosgenetics*2Fs*2Fdata-availability/1/010f0195e60178a4-69d20206-bd1d-45da-953c-46d80280729c-000000/FCm3JHmVbZ8xu2q1lIXfLFB3fGrmo39Dj4lv2dbJl0g=204__;JSUlJSU!!PDiH4ENfjr2_Jw!ERL3sGoc9UO92cr3T_cWb1kk1_8Ts9dUbJDVCXIE6qhGMqpbO7iluMZgjI0IKCe6VA4CZ6enomLE_tat_vgDHRvlaWLwTEm_mhii8w$), and numerical data that underlies graphs or summary statistics should be provided in spreadsheet form as supporting information.

Reviewer #1: **No:** Results of RNA-seq and ChIP-seq experiments have not been provided as supplemental files

Reviewer #2: **No:** RNAseq and ChIPseq datasets presented in this study do not have a public repository accession number provided. It is highly recommended that these datasets are made publicly available prior to publication. Supplying uncropped western blot images as supplementary figures is also highly recommended.

Reviewer #3: Yes

The Datasets are deposited on GEO with restricted access; public access will be available upon publication. Minimal datasets have been included in the Supporting Information files.
