# Supplementary material for: Epigenome and transcriptome changes in KMT2D-related Kabuki syndrome Type 1 iPSCs, neuronal progenitors and cortical neurons
Source: PLoS Genet. 2025 Sep 19;21(9):e1011608. doi: 10.1371/journal.pgen.1011608 (PMC12468740; doi:10.1371/journal.pgen.1011608)
Supplement: Attachment — Submitted filename: Reviewers comments PLoS Genetics - 2nd round.docx [file pgen.1011608.s027.docx]

Response to Referees’ comments

Epigenome and transcriptome changes in KMT2D-related Kabuki syndrome Type 1 iPSCs, neuronal progenitors and cortical neurons

We thank the referees and editor for their constructive comments. All changes have been added and highlighted in red and deleted text is tracked throughout the manuscript.

**Journal Requirements:**

Please amend your detailed Financial Disclosure statement. This is published with the article. It must therefore be completed in full sentences and contain the exact wording you wish to be published. Please ensure that the funders and grant numbers match between the Financial Disclosure field and the Funding Information tab in your submission form. Note that the funders must be provided in the same order in both places as well.

State what role the funders took in the study. If the funders had no role in your study, please state: "The funders had no role in study design, data collection and analysis, decision to publish, or preparation of the manuscript.".

If you did not receive any funding for this study, please simply state: u201cThe authors received no specific funding for this work.u201d

We have attended to this.

**Reviewers' comments:**

**Reviewer #1**: All of my comments have been thoroughly addressed. The authors have done a clear and thorough job of addressing all points.

**Reviewer #3:** Unfortunately, several of the most critical comments remain inadequately addressed in the rebuttal, leaving key concerns unresolved and diminishing confidence in the rigor and interpretability of the study. While the authors provided responses, many of them either sidestepped the core issues or deferred them to future work without proper justification.

1a. In this study, the authors performed transcriptomic and epigenetic profiling of iPSC-derived

cell models (iPSCs, neuronal progenitors, and neurons) to investigate molecular changes in

KS1. They utilized three different iPSC lines from KS1 patients. However, the core analyses

were conducted using only one KS1 iPSC line with a missense mutation (c.16019G>A,

p.(Arg5340Gln)), making it difficult to generalize the proposed mechanism (Polycomb mediated repression and transcription factor binding changes).

The decision to use only one non-isogenic control remains highly problematic and random, especially in a context where genetic background is a known confounder. The authors have not added any substantial clarification or justification for this design choice. The authors should at least incorporate a dedicated paragraph in the Discussion to highlight the significant limitations imposed by their case-control design. As it stands, the study lacks the statistical power and experimental design required to support any strong or definitive conclusions.

We thank the reviewer for their additional comments and suggestions.

In terms of justification for our experimental design choice of performing most of the work on the missense variant we have stated in the manuscript that most previous studies have used KS models with null variants (lines 78-79) and we wanted to address this gap.

To enhance the statistical power of or sudy, we have now expanded the RNASeq validation by including RNASeq data from Day 0 and Day 30 in all three KS1 lines and three control lines (Figure 6).

As suggested, we have now included a dedicated paragraph (lines 382-385) in the Discussion section highlighting the limitations posed by our study design,

1b. iPSC characterisation informations

Details regarding the control setup should be clearly specified in the Materials and Methods section.

We have included the required information in the Material and Methods section (line 434-435).

1c. Figure 5G: The overlap of differentially expressed genes (DEGs) between the three KS1

iPSC lines is only briefly explored, and only between iPSCs and neuronal progenitors. The

observed overlap between all three lines is of high interest, as these likely represent the most

robust KMT2D-dependent changes. However, the presented data are sparse and feel

incomplete, with no corresponding methylation data.

The authors' response that further analysis will be part of future work is not acceptable in this context. They already have the data available, and given the limitations of their case-control setup, it is crucial to robustly identify differentially expressed genes (DEGs) within the current study. This is not a matter for future investigation but a requirement for the current manuscript to meet basic standards of scientific rigor.

We thank the reviewer for his comment. We have now included new validation data and performed differential analysis for all the lines and all three stages (see new Figure 6) .

2. No Orthogonal Validation Performed

There is still no orthogonal validation (e.g., immunocytochemistry) of the key findings. The authors suggest this could be explored in future work. Validating altered pathways through straightforward immunocytochemical approaches should be part of the present analysis, not deferred.

We appreciate the reviewer comment but unfortunately, this beyond the scope of our current project.

3. fig 1D Unclear Representation of Western Blot Data

Regarding Figure 1d, the authors assert that three controls were used and Western blots were performed in triplicate. Yet only three data points are shown, raising doubts about whether all replicates are properly represented. The explanation provided fails to clarify this issue, especially given the substantial variation evident on the blots. To address this properly, the authors should display the individual replicates for each line and report statistics per line rather than presenting pooled averages. This is a basic expectation for transparent and interpretable data reporting

Experiments were performed in triplicate (or more) for each cell lines. The initial presentation reflected averaged values per line, leading to an apparent count of three data points. This may give misleading interpretation on transparency of our results. To address this, we have now revised the data presentation to include all nine or more individual replicates across each samples. Additionally, we have performed more replicates for the H3K4me3 mark as there were some outlier values from one western blot (A), which have now been removed (B).

A	B
